# Standards and pitfalls of focal ischemia models in spontaneously hypertensive rats: With a systematic review of recent articles

**DOI:** 10.1186/1479-5876-10-139

**Published:** 2012-07-06

**Authors:** Hiroshi Yao, Toru Nabika

**Affiliations:** 1 Laboratory for Neurochemistry, Center for Emotional and Behavioral Disorders, National Hospital Organization Hizen Psychiatric Center, Mitsu 160, Yoshinogari, Kanzaki, Saga, 842–0192, Japan; 2 Department of Functional Pathology, Shimane University School of Medicine, Izumo, Japan

**Keywords:** Focal ischemia, Hypertension, Experimental, Genetics, Animal Models, Cerebrovascular disease, Stroke

## Abstract

We reviewed the early development of various focal ischemia models in spontaneously hypertensive rats (SHR), and summarized recent reports on this topic. Among 6 focal ischemia models established in divergent substrains of SHR, distal middle cerebral artery occlusion is the most frequently used and relevant method of focal ischemia in the light of penumbra concept. We performed an online *PubMed* search (2001–2010), and identified 118 original articles with focal ischemia in SHR. Physiological parameters such as age, body weight, and even blood pressure were often neglected in the literature: the information regarding the physiological parameters of SHR is critical, and should be provided within the methodology section of all articles related to stroke models in SHR. Although the quality of recent studies on neuroprotective strategy is improving, the mechanisms underlying the protection should be more clearly recognized so as to facilitate the translation from animal studies to human stroke. To overcome the genetic heterogeneity in substrains of SHR, new approaches, such as a huge repository of genetic markers in rat strains and the congenic strategy, are currently in progress.

## Introduction

Almost 50 years have passed since the spontaneously hypertensive rats (SHR) were established [1]. In the present review, we will focus on the early development of focal ischemia models in SHR, and provide a critical systematic review on recent reports on SHR stroke models. SHR are one of the most widely used genetic models for hypertension. Hypertension is a major risk factor for stroke and most other cardiovascular diseases, and therefore SHR are relevant to stroke research. SHR were initially obtained by selective inbreedings from the Wistar-Kyoto rats (WKY) with the highest blood pressure. The stroke prone SHR (SHRSP) were established from the A substrain of the SHR, and the other 2 substrains (B and C) are resistant to spontaneous stroke [2]. The B substrain corresponds to SHR/Izm (Izumo). SHR were sent to the National Institute of Health (NIH) at the F13 generation in 1966.

The advantages of using SHR in contrast to normotensive rats in stroke research are: (1) presence of comorbidity (i.e., hypertension), (2) reproducible and adequate-sized infarction after distal middle cerebral artery occlusion (MCAO) alone, and (3) a similar therapeutic time window and cerebral blood flow (CBF) threshold for infarction to normotensive rats [3,4]. Shortcomings are: (1) SHR and SHRSP are expensive, (2) high mortality in aged SHR and SHRSP, and (3) resistance to therapy. Another confounding problem with using SHR in stroke research is that SHR and WKY from different sources are genetically heterogeneous [5-7]. Because WKY are rarely used for focal ischemia models or their controls in recent years, we do not mention detailed descriptions of genetic heterogeneity of WKY.

### Stroke proneness and stroke sensitivity

Focal ischemia models are usually based on MCAO and mimic human acute brain infarction caused by occlusion of major cerebral arteries. Small vessel disease is another subtype of stroke, and is a major cause of vascular cognitive impairment. Spontaneous stroke in SHRSP, often under salt loading, is a unique and relevant feature of SHRSP. The most preponderant site for spontaneous stroke in SHRSP was the cortical area supplied by the anterior and posterior cerebral arteries with recurrent branching [8]. A systematic review summarized that animals sacrificed after developing stroke-like symptoms displayed arteriolar wall thickening, subcortical lesions, enlarged perivascular spaces and cortical infarcts and hemorrhages [9]. Hainsworth and Markus [10] concluded that to model small vessel disease-like arteriopathy, SHRSP appears closest to human small vessel disease, particularly in aged animals. In salt-loaded SHRSP, the number of days necessary to develop stroke has been used as an index of stroke proneness [11,12]. In contrast, infarct volume after MCAO is an index of stroke sensitivity. Tamura et al. described the first surgical MCAO in the rat [13]. Subsequently, the importance of hypertension was emphasized in focal ischemia models [14-16]. Both SHR and SHRSP have increased stroke sensitivity to focal ischemia (i.e., larger infarct size after MCAO) compared with normotensive rats [17]. Increased stroke sensitivity is likely attributable to not only spontaneous hypertension but also additional genetic factors that will be discussed later. Many neuroprotective strategies in stroke research are based on the penumbra concept constructed using focal ischemia models [18]. In this context, focal ischemia models are relevant to the human clinical setting of ischemic stroke or brain infarction.

### Focal ischemia models in SHR

#### Distal MCAO

**Figure 1:**
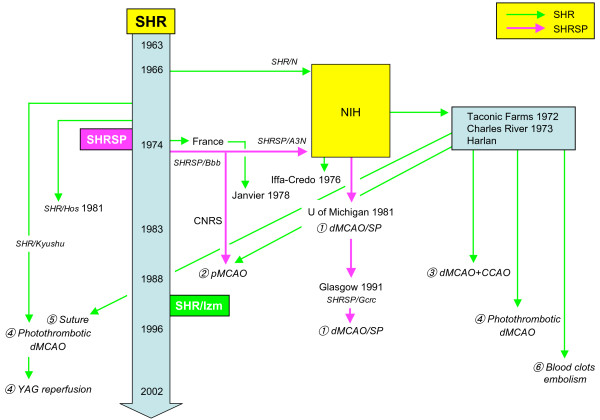
** Focal ischemia models in divergent substrains of spontaneously hypertensive rats (SHR).** SHR were sent to National Institute of Health (NIH) at the F13 generation in 1966 (SHR/N), and then from NIH to Charles River, Taconic Farms, and Harlan. In France, Iffa-Credo received their original breeding stocks of SHR from NIH in 1976, and Janvier started breedings in 1978 with SHR derived from the Delalande Research Center, which in turn received their original breeding stocks from Kyoto University in 1974. Two colonies of SHRSP (SHRSP/A3N [NIH] and SHRSP/Bbb [Heidelberg]) were separated at F35, 36 in 1975. CNRS, Centre National de la Recherche Scientifique; SHRSP or SP, stroke prone SHR: NIH, National Institute of Health; MCAO, middle cerebral artery occlusion; d, distal; p, proximal; CCAO, common carotid artery occlusion; YAG, yttrium-aluminium-garnet (laser).

Six focal ischemia models have been established in SHR and SHRSP (Figure 1). SHRSP maintained at the University of Michigan originated from the NIH stock in 1981 [19]. Using SHRSP/Michigan, the pathophysiological consequences of MCAO distal to striate branches were studied for the first time in SHRSP [14]. Even in young SHRSP without chronic hypertension, distal MCAO produced larger lesions than WKY or Sprague–Dawley rats. This approach of Coyle was later employed by Fujii et al. to investigate the beneficial effects of chronic antihypertensive treatment on infarct size after MCAO: systolic blood pressure in SHRSP was reduced to the level of WKY with antihypertensive treatment after 3 months of age for 3 months, which reduced infarct volume by approximately 40% compared with untreated SHRSP [20]. The inbred colonies of SHRSP, maintained in the Department of Medicine and Therapeutics at the University of Glasgow (SHRSPGla or SHRSP/Gcrc) since 1991, were derived from the colony of University of Michigan. 

#### Proximal MCAO

The approach of Tamura et al., which occludes middle cerebral artery (MCA) proximal to the lenticulostriate arteries, produces infarction of both the cortex and the lateral part of striatum [13]. Duverger and MacKenzie employed the modified method of Tamura et al., using SHRSP (*Centre National de la Recherche Scientifique* [CNRS], France), SHR (Charles River, Italy), and 3 normotensive strains (WKY, Sprague-Dawley, and Fischer-344) [15]. In hypertensive rats, MCAO resulted in a considerable volume of infarction (158.1 ± 4.4 [SEM] mm^3 ^in SHR and 172.4 ± 4.4 [SEM] mm^3 ^in SHRSP), and the variability was minimum (coefficient of variation [C.V.] = 7-8%) compared with the 3 normotensive strains (C.V. = 20-49%). In the normotensive control WKY, the infarction was small (63.2 ± 10.9 [SEM] mm^3^).

#### Tandem MCAO

Brint et al. summarized their experience in 3 strains of rats (SHR/Charles River or Taconic Farms, Wistar, and Fisher-344 rats) with focal ischemia produced by tandem occlusion of the distal MCA and ipsilateral common carotid artery [16]. Of the 3 strains of rats, the SHR showed the largest (157–259 mm^3^) and most reproducible (average C.V. = 19%) infarctions, and statistical power analysis revealed that tandem occlusion of the distal MCA and ipsilateral common carotid artery in the SHR strain offered a practical model in terms of requisite animals necessary to avoid a Type 2 error (false negative) for differences in infarct volume between control and experimental groups. Although infarct volume might be more reproducible in SHR with tandem carotid artery occlusion, the amount of penumbra tissue may also be reduced. Consequently, it may be more difficult to demonstrate neuroprotective efficacy in these models with tandem occlusion.

#### Photothrombotic MCAO

In the context of thrombosis, the effects of thrombotic stroke on compromised brain tissue may be different from those due to cerebral ischemia induced by mechanical occlusions of intracranial or extracranial brain arteries [21]. Prado et al. first used SHR as a photothrombotic distal MCAO model, avoiding common carotid artery involvement [22]. SHR/Kyushu and later SHR/Izm were employed for photothrombotic distal MCAO [23,24]. The SHR/Kyushu were from the F20 and F21 generations derived by Okamoto and Aoki [25]. A photothrombotic distal MCAO model in SHR yields a highly reproducible infarct volume (average C.V. = 21%) and does not entail extensive surgery or opening of the dura, thereby avoiding unacceptable local tissue trauma at the site of MCAO [17]. This model encompasses appropriate physiological monitoring, associated risk factors for stroke, and clinically relevant pathophysiology of thrombosis. Cai et al. showed the infarct size was larger in male and female SHR/Kyushu than in SHR/Izm (i.e., substrain differences) [24]. Because blood pressure levels were the same between the two substrains, factors other than hypertension probably account for different lesion size. The ultraviolet laser–induced reperfusion method, indicated as YAG reperfusion in Figure 1, was also achieved by Watson et al., [26] which was applied to SHR/Kyushu [23] and later to SHR/Izm [27].

#### Intraluminal suture occlusion

The intraluminal suture model, developed by Koizumi et al. [28] and Longa et al. [29], is undoubtedly the most frequently used focal ischemia model in rats and mice [30]. To our knowledge, Kawamura et al. first adopted SHR for intraluminal suture model followed by a number of experiments with this method [31]. Alkayed et al. investigated gender-linked brain injury in experimental stroke produced by intraluminal suture occlusion in SHRSP maintained in Johns Hopkins University from a stock obtained from the NIH [32]. Some of the major advantages of the intraluminal suture model are that it is easy to perform, minimally invasive, and most importantly does not require craniectomy. However, it appears that SHR can be replaced with normotensive rats in many experiments with the use of the intraluminal suture occlusion method except for the studies on antihypertensive agents.

Although reperfusion of ischemic brain tissue is critical for restoring normal function, it can paradoxically result in secondary damage or reperfusion injury. Several lines of evidence suggest that post-ischemic oxidative stress and inflammation contribute to brain injury [33]. As reperfusion injury is the condition of ischemia aggravated by the occurrence of reperfusion more so than in the case where reperfusion does not occur (i.e., permanent occlusion), in order to see reperfusion injury it is necessary to make a comparison with permanent occlusion. From a critical point of view, the intraluminal suture model is a top of the internal carotid artery occlusion model rather than a MCAO model [34]. Consequently, this model has a wide ischemic zone, and because the mortality rate is high in the case of permanent occlusion, the permanent occlusion group and the reperfusion group that shared the same time lapse cannot be compared.

The intraluminal suture model in SHR had a high incidence of parenchymal hematomas, and therefore is appropriate for the evaluation of reperfusion-associated hemorrhagic transformation [35]. Hemorrhagic transformation is one of the ultimate forms of reperfusion injury. SHR subjected to 3 h of transient suture MCAO had significant vascular injury or hemorrhagic infarction compared to normotensive rats [36]. Yamashita et al. demonstrated that tPA administered just before the reperfusion of 4.5 h suture MCAO induced dissociation of the neurovascular unit (i.e., the detachment of astrocyte endfeet from the basement membrane), which was prevented by a free radical scavenger, edaravone [37].

#### Blood clot embolism

Although fibrinolytic therapy with tissue plasminogen activator (tPA) is effective in the treatment of acute stroke, there is an elevated risk of brain hemorrhage [38,39]. Hypertension is one of the factors related to the increased incidence of hemorrhagic complications after tPA treatment [40]. A novel model of tPA-induced hemorrhage was examined in an embolic focal ischemia model with homologous blood clots in SHR [41]. Blood pressure was considered to be a critical correlate, because tPA-induced extensive hemorrhagic transformation was observed in SHR but not in normotensive WKY. Since reduction of blood pressure with hydralazine, administered in the drinking water for 1 week before MCAO, significantly reduced the incidence of hemorrhagic transformation, elevated blood pressure during tPA-induced reperfusion is considered to contribute to the pathogenesis of hemorrhage [42]. Therefore, SHR are relevant as a model of embolic stroke as they demonstrate hemorrhagic transformation after tPA therapy.

In the SHR clot embolism model, one study showed a reduction of secondary hemorrhage after thrombolysis combined with normobaric and hyperbaric oxygen therapy [43], while normobaric oxygen did not interfere with the beneficial action of tPA in another study [44].

### Penumbra and the SHR stroke models

**Figure 2:**
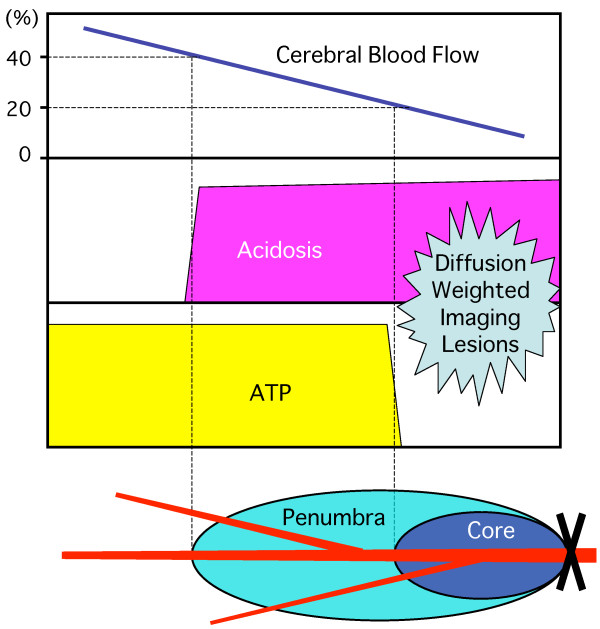
** Schematic representation of the ischemic penumbra.** Glucose utilization (anaerobic glycolysis) transiently increases at a CBF value below about 40% of normal, which corresponds to the beginning of acidosis with lactate accumulation. At a CBF value below about 20% of normal, a breakdown of energy state begins with reduced adenosine triphosphate (ATP), resulting in the condition of ischemic core. The penumbra is defined as an area in which metabolism is impaired due to reduced CBF, but the cellular polarization is still maintained without diffusion- weighted imaging lesions on magnetic resonance imaging. Therefore, an acidosis/diffusion- weighted imaging mismatch represents penumbra.

Penumbra, analogous to the half-shaded zone around a solar eclipse, is the zone of salvageable tissue around the ischemic core (Figure 2). The classic concept of ischemic penumbra is defined as the condition of an ischemic brain with CBF between the upper threshold of electrical silence and the lower threshold of energy and ion pump failure [18,45]. Regions having CBF in the ischemic core range (CBF 0% to 20% of control values) had a 96% probability of undergoing infarction, and zones of higher CBF (>40% of control) were largely spared from infarction [46]. One of the most sensitive biochemical markers of cerebral ischemia is the inhibition of protein synthesis, which occurs above the upper threshold of classical penumbra. Under experimental conditions, the most reliable way to localize the ischemic core is the loss of adenosine triphosphate (ATP), and the biochemical marker of the core plus penumbra is tissue acidosis [47]. Therefore, pH-weighted magnetic resonance imaging and diffusion-weighted imaging mismatch can provide a more comprehensive zone of penumbra [48]. In the clinical routine, however, perfusion-weighted imaging/diffusion-weighted imaging mismatch is used as a surrogate marker for the penumbra: the early diffusion-weighted imaging lesion might define the ischemic core, while the adjacent critically hypoperfused tissue might be identified as penumbra with perfusion-weighted imaging [49]. Even after early reperfusion with tPA, the sustained reversal of diffusion abnormality was minimal, which indicates that the infarct core is well represented by the acute diffusion lesion [50]. The linear increase in brain sodium after MCAO, which indicates ischemic core, was demonstrated directly by sodium magnetic resonance imaging, and therefore sodium magnetic resonance imaging could provide a better measure of tissue viability compared to diffusion-weighted imaging [51]. 

It has not been clear, however, which of the focal ischemia models in SHR is most relevant to the penumbra concept. Proximal MCAO produces two ischemic vascular territories of lenticulostriate arteries and cortical branches of MCA, the former of which are end-arteries and therefore treatment-resistant with little penumbra. The intraluminal suture model is a type of internal carotid artery occlusion model rather than a pure MCAO model [34]. In clinical settings, the arterial obstruction site strongly predicts infarct growth and clinical outcome: internal carotid artery occlusion carried a uniformly poor prognosis with poor response to tPA therapy [52]. Although the infarcts produced by distal MCAO in normotensive rats are small with presumably limited size of penumbra, distal MCAO alone in SHR results in substantial size of infarction. However, genetic hypertension (i.e., SHR) and induced renovascular hypertension resulted in a larger lesion and smaller penumbra compared with normotensive WKY after intraluminal suture occlusion [53]. In the case of combined carotid artery occlusions, concurrent carotid occlusions may excessively suppress the retrograde collateral flow to the penumbra. Taken together, according to the ischemic penumbra concept, the distal MCAO model without carotid artery involvement is the best choice for human stroke (i.e., one major cerebral artery occlusion with collateral perfusion) among SHR stroke models (Figure 2).

In our experience with distal MCAO in SHR, the early restoration of perfusion confers advantages on the ischemic brain at risk. The cortical region, rescued by early reperfusion, was considered to be the penumbral zone [54]. At 6 h after MCAO, lesion volume (not infarction), determined with 2,3,5-triphenyltetrazolium chloride (TTC), was the same among the groups of permanent, 1 h, and 2 h MCAO, whereas 2 h and permanent MCAO produced larger infarction than 1 h MCAO by approximately 2-fold after 24 h to 48 h [27]. Although TTC methods underestimate the infarct volume because of reactive gliosis, macrophage infiltration, and other proliferative responses in the infarction at later time points [55], TTC staining is a convenient procedure and reliable for detection of brain infarction at 24 h after the onset of ischemia [56]. Hence, the volume of penumbra is considered to be approximately 50% of final infarct volume in our model. In the ischemic core, Na^+ ^increased and K^+ ^decreased progressively 3 h to 6 h after onset of ischemia, while these electrolytes were within normal values in the acute phase of brain ischemia in the penumbral zone [57]. In this stroke model, apoptotic internucleosomal cleavage (DNA ladder), which is energy dependent, was observed in the penumbral zone but not in the ischemic core at 6 h after MCAO, which was preceded by large DNA fragmentation in both penumbra and core at 3 h after MCAO [58]. Protein synthesis is inhibited at a CBF value clearly above the disturbance of glucose utilization and energy metabolism. Attenuation or recovery of deranged proteomic profile was slow even after a short period of ischemia (i.e., 1 h MCAO). Nevertheless, it was not permanently disturbed in the reperfused-penumbra, while a derangement in proteomic profile appeared to be an irreversible process in the ischemic core [59]. These results also support the view that the distal MCAO model in SHR provides a rational approach to the penumbra concept.

### MCAO experiments in SHR: A systematic review

We performed an online *PubMed* search based on the terms “spontaneously hypertensive rats AND focal ischemia” with limits “English, and Animal” and analyzed all original articles published between 2001 and 2010. We also hand-searched 2 journals (*Stroke* and *Journal of Cerebral Blood Flow and Metabolism*) that had published a large proportion of relevant material. We identified 118 original articles dealing with focal ischemia in SHR and SHRSP (Table 1). Stroke resistant or regular SHR were used in 109 of 118 experiments (92%). Recently, SHR have mostly been obtained from commercial suppliers such as Charles River (SHR/NCrl), Taconic Farms (SHR/NTac), Harlan (SHR/NHsd), Janvier, and the Disease Model Cooperative Research Association or Japan SLC (SHR/Izm) (80 of 118 experiments [68%]), and SHR 19 (16%) were from research institutes. Models of distal MCAO, distal MCAO combined with ipsilateral common carotid artery occlusion, and photothrombotic distal MCAO were all based on the occlusion of distal portion of MCA: distal MCAO is the predominant method (55 of 118 experiments [47%]) of focal ischemia in SHR. The intraluminal suture model was also frequently used (42 of 118 experiments [36%]) as expected.

**Table 1:** Focal ischemia models in spontaneously hypertensive

**Author**	**Substrains**	**Age**	**BW (g)**	**MABP (mmHg)**	**Methods of MCAO**	**CBF**	**References**
Oyama N, et al.	SHR/Charles River Jpan	10 wk	NA	124±5 (SEM)	intraluminal suture	LDF	J Neurosci Res 2010;88:2889-2898
Zhao X, et al.	SHR/Harlan	NA	NA	NA	dMCAO+ipsi-CCAO (Brint)	LDF	Stroke 2010;41:363-367
Kranz A, et al.	SHR/Charles River	NA	250	NA	pMCAO (Tamura)		Brain Res 2010;1315:128-136
Yang Y, et al.	SHR*	NA	300-320	NA	intraluminal suture		J Neurochem 2010;112:134-149
Ito H, et al.	SHR/Hos, Japan SLC	20-24 wk	346±33	209±13	photothrombotic dMCAO	LDF	J Cereb Blood Flow Metab 2010;30:343-351
Sun L, et al.	SHR/Janvier France	NA	300-350	120±11	blood clots embolism		J Cereb Blood Flow Metab 2010;30:1651-1660
Porritt MJ, et al.	SHR/ARC Australia	16 mo	438±11	SBP=200-210	intraluminal suture		J Cereb Blood Flow Metab 2010;30:1520-1526
Yao H, et al.	SHR/Izm, Japan SLC	5-7 mo	352±12	172±12	photothrombotic dMCAO/Re	LDF	Neurochem Res 2009;34:1999-2007
Yao H, et al.	SHR/Izm, Japan SLC	5-7 mo	NA	182 (mean)	photothrombotic dMCAO/Re	LDF	J Cereb Blood Flow Metab 2009;29:565-574
McCabe C, et al.	SHRSP/Gla	12-16 wk	220-390	93±4	intraluminal suture	MRI	Stroke 2009;40:3864-3868
Yan Y-P, et al.	SHR/Charles River USA	NA	270-320	NA	intraluminal suture	LDF	Neurochem Int 2009;55:826-832
Ashioti M, et al.	SHR/Charles River UK	NA	200-250	NA	dMCAO+ipsi-CCAO		BMC Neurosci 2009;10:82
Ishikawa E, et al.	SHR/Kyushu	5-8 mo	360-440	154±11	photothrombotic dMCAO	LDF	J Neurol Sci 2009;285:78-84
Omura-Matsuoka E, et al.	SHR/Charles River Jpan	10-15 wk	250-350	181±10	intraluminal suture	LDF	Hypertens Res 2009;32:548-553
Cao F, et al.	SHRSP*	13-15 wk	250-300	NA	dMCAO		Brain Res 2009;1272:52-61
Liu Y-P, et al.	SHR/Charles River USA	NA	290-310	130-140	intraluminal suture	LDF	J Cereb Blood Flow Metab 2009;29:780-791
Sasaki T, et al.	SHR/Charles River Jpan	NA	320±40	NA	dMCAO+ipsi-CCAO (Brint)	LDF	Neurosci Lett 2009;449:61-65
Henning EC, et al.	SHR/Charles River USA	3-4 mo	350±20	NA	intraluminal suture		J Cereb Blood Flow Metab 2009;29:1229-1239
Dharap A, et al.	SHR/Charles River USA	NA	280-300	NA	intraluminal suture		J Cereb Blood Flow Metab 2009;29:675-687
Ishibashi S, et al.	SHR/Charles River USA	8 wk	180-220	NA	dMCAO		J Cereb Blood Flow Metab 2009;29:606-620
Yamashita T, et al.	SHR/Izm, Japan SLC	13 wk	250-280	117±29	intraluminal suture	LDF	J Cereb Blood Flow Metab 2009;29:715-725
Fujiwara N, et al.	SHR/Charles River USA	NA	303±8	183±6	blood clots embolism	LDF	BMC Neurosci 2009;10:79
Murata Y, et al.	SHR/Charles River USA	NA	283±8	184±5	blood clots embolism	LDF	Stroke 2008;39:3372-3377
Matsuzaki T, et al.	SHR/Izm, Japan SLC	9 wk	240-290	126±17	intraluminal suture	LDF	Neurol Res 2008;30:531-535
Kozak W, et al.	SHR/Charles River USA	12-13 wk	275-300	135±8 (telemetry)	intraluminal suture	LDF	J Pharmacol Exp Ther 2008;326:773-782
Henning EC, et al.	SHR/Charles River USA	3-4 mo	300-360	SBP=165-185	intraluminal suture		Stroke 2008;39:3405-3410
Park S-W, et al.	SHR/Charles River USA	NA	250-350	NA	intraluminal suture	LDF	J Neurosurg 2007;107:593-599
Korde AS, et al.	SHR/Harlan	NA	250-350	94±3	dMCAO+ipsi-CCAO (Brint)	LDF	J Neurotrauma 2007;24:895-908
Kurasako T, et al.	SHR/Harlan	NA	220-300	101±12	dMCAO+ipsi-CCAO (Brint)		J Cereb Blood Flow Metab 2007;27:1919-1930
Jimenez-Altayo F, et al.	SHR/Janvier Spain	13-15 wk	341±4 (SEM)	SBP=204±2 (SEM)	intraluminal suture		Am J Physiol Heart Circ Physiol 2007;293:H628-635
Tureyen K, et al.	SHR/Charles River USA	NA	NA	135±9	intraluminal suture	LDF	J Neurochem 2007;101:41-56
Yao H, et al.	SHRSP/Izm, Japan SLC	5 mo	315±30	241±22	photothrombotic dMCAO	LDF	Physiol Genomics 2007;30:69-73
Ashioti M, et al.	SHR/Charles River UK	NA	265±9	NA	dMCAO+ipsi-CCAO		Brain Res 2007;1145:177-189
Mariucci G, et al.	SHR/Charles River Italy	12-14 wk	250-330	NA	pMCAO (Tamura)		Neurosci Lett 2007;415:77-80
Adibhatla RM, et al.	SHR/Charles River USA	NA	250-300	NA	intraluminal suture		Brain Res 2007;1134:199-205
Leker RR, et al.	SHR/Tel Aviv	NA	NA	NA	dMCAO		Stroke 2007;38:153-161
Kumai Y, et al.	SHR/Kyushu	5-8 mo	340-435	164±2 (SEM)	photothrombotic dMCAO	LDF	J Cereb Blood Flow Metab 2007;27:1152-1160
Yang Y, et al.	SHR*	NA	300-320	NA	intraluminal suture		J Cereb Blood Flow Metab 2007;27:697-709
Yan Y-P, et al.	SHR/Charles River USA	NA	270-300	NA	intraluminal suture	LDF	J Cereb Blood Flow Metab 2007;27:1213-1224
Takasawa M, et al.	SHR/Charles River UK	NA	295-305	NA	dMCAO+ipsi-CCAO		J Cereb Blood Flow Metab 2007;27:679-689
Akaiwa K, et al.	SHR*	NA	250-290	144±5	intraluminal suture	LDF	Brain Res 2006;1122:47-55
Yan Y-P, et al.	SHR/Charles River USA	NA	250-300	130-140	intraluminal suture	LDF	Eur J Neurosci 2006;24:45-54
Lammer A, et al.	SHR/Charles River Germany	NA	250-300	NA	pMCAO (Tamura)		Eur J Neurosci 2006;23:2824-2828
Bowen KK, et al.	SHR/Charles River USA	NA	280-320	NA	intraluminal suture	LDF	Neurochem Int 2006;49:127-135
Komitova M, et al.	SHR/Mollegaard	6 mo	NA	NA	dMCAO		Exp Neurol 2006;199:113-121
Zhao L, et al.	SHR/Charles River USA	NA	250-350	118±12	dMCAO+ipsi-CCAO (Brint)	AR / LDF	J Cereb Blood Flow Metab 2006;26:1128-1140
Zhang B, et al.	SHRSP*	16-18 wk	NA	204±7	dMCAO		J Cereb Blood Flow Metab 2006;26:708-721
Furuya K, et al.	SHR/Charles River Jpan	NA	280-320	NA	dMCAO+ipsi-CCAO (Brint)	LDF	J Neurosurg 2005;103:715-723
Kamiya T, et al.	SHR/Harlan	NA	225-325	161±13	dMCAO+ipsi-CCAO (Brint)	AR	Stroke 2005;36:2463-2467
Naylor M, et al.	SHR/Charles River USA	NA	280-320	NA	intraluminal suture	LDF	Neurochem Int 2005;47:565-572
Adibhatla RM, et al.	SHR*	NA	250-275	NA	intraluminal suture	LDF	Brain Res 2005;1058:193-197
Tsuji K, et al.	SHR*	NA	260-280	NA	intraluminal suture	LDF	Stroke 2005;36:1954-1959
Gunther A, et al.	SHR/Charles River Germany	NA	250-330	NA	pMCAO (Tamura)		Eur J Neurosci 2005;21:3189-3194
Vemuganti R	SHR/Charles River USA	NA	280-320	NA	intraluminal suture	LDF	Neurochem Int 2005;47:136-142
Komitova M, et al.	SHR/Mollegaard	6 mo	300-370	NA	dMCAO		Eur J Neurosci 2005;21:2397-2405
Onoue S, et al.	SHRSP*	NA	280-320	NA	dMCAO+ipsi-CCAO (Brint)		Brain Res Mol Brain Res 2005;134:189-197
Hobohm C, et al.	SHR/Charles River Germany	NA	250-330	NA	pMCAO (Tamura)		J Neurosci Res 2005;80:539-548
Drummond JC, et al.	SHR/Harlan	16-20 wk	375-425	131±9	intraluminal suture		Anesth Analg 2005;100:841-846
Ooboshi H, et al.	SHR/Kyushu	5-8 mo	340-430	NA	photothrombotic dMCAO	LDF	Circulation 2005;111:913-919
Takada J, et al.	SHR/Kyushu	7-8 mo	352-450	153±10	photothrombotic dMCAO	LDF	Gene Ther 2005;12:487-493
Kumai Y, et al.	SHR/Kyushu	5-10 mo	320-400	181±21	photothrombotic dMCAO	LDF	J Cereb Blood Flow Metab 2004;24:1359-1368
Carswell HV, et al.	SHRSP/Gla, female	3-4 mo	179±11	140±15	dMCAO		J Cereb Blood Flow Metab 2004;24:298-304
Franke H, et al.	SHR/Charles River Germany	NA	250-330	NA	pMCAO (Tamura)		J Neuropathol Exp Neurol 2004;63:686-699
Zhang B, et al.	SHRSP*	12-13 wk	250-300	NA	dMCAO		Neuroscience 2004;126:433-440
Dhodda VK, et al.	SHR/Charles River USA	NA	280-320	NA	intraluminal suture	LDF	J Neurochem 2004;89:73-89
Nurmi A, et al.	SHR/M&B A/S	NA	NA	NA	pMCAO		Stroke 2004;35:987-991
Vemuganti R, et al.	SHR/Charles River USA	NA	280-320	124±11	intraluminal suture	LDF	Stroke 2004;35:179-184
Ren Y, et al.	SHR/Harlan	NA	250-300	124±16	dMCAO+ipsi-CCAO (Brint)		J Cereb Blood Flow Metab 2004;24:42-53
Kumai Y, et al.	SHR/Kyushu, female	5-7 mo	195-240	170±2 (SEM)	photothrombotic dMCAO	LDF	Exp Neurol 2003;184:904-911
Babu GN, et al.	SHR*	NA	250-300	NA	intraluminal suture	LDF	Neurochem Res 2003;28:1851-1857
Gautier S, et al.	SHR/Elevage Janvier France	NA	270-320	NA	intraluminal suture		Stroke 2003;34:2975-2979
Asahi M, et al.	SHR/Taconic Farms	NA	NA	NA	blood clots embolism	LDF	J Cereb Blood Flow Metab 2003;23:895–899.
Teichner A, et al.	SHR*	13 wk	NA	NA	dMCAO		Exp Neurol 2003;182:353-360
Sadanaga-Akiyoshi F, et al.	SHR/Kyushu	5-7 mo	315-425	178±11/176±7	photothrombotic dMCAO	LDF	Neurochem Res 2003;28:1227-1234
Dahlqvist P, et al.	SHR/Mollegaard	14-15 wk	NA	NA	dMCAO		Neuroscience 2003;119:643-652
Pettigrew LC, et al.	SHR/Harlan	NA	250-300	98±17	dMCAO+ipsi-CCAO (Brint)		Neurol Res 2003;25:201-207
Yan Y, et al.	SHR/Charles River USA	NA	270-310	133±9	intraluminal suture		Brain Res 2003;961:22-31
Yamakawa H, et al.	SHR/Taconic Farms	12 wk	300-500	SBP=156±4	dMCAO		J Cereb Blood Flow Metab 2003;23:371-380
Wiessner C, et al.	SHR/Iffa Credo	NA	220-300	NA	dMCAO		J Cereb Blood Flow Metab 2003;23:154-165
Rao VLR, et al.	SHR/Charles River USA	NA	280-320	NA	intraluminal suture	LDF	J Neurochem 2002;83:1072-1086
Aoki T, et al.	SHR*	NA	NA	177±6	blood clots embolism	LDF	Stroke 2002;33:2711-2717
Leker RR, et al.	SHR/Tel Aviv	13 wk	NA	127±11	dMCAO	LDF	Exp Neurol 2002;176:355-363
Leker RR, et al.	SHR/Tel Aviv	13 wk	NA	127±5	dMCAO	LDF	Stroke 2002;33:1085-1092
Adibhatla RM, et al.	SHR*	NA	NA	NA	intraluminal suture		Brain Res 2002;938:81-86
Sakakibara Y, et al.	SHR/Charles River USA	NA	280-350	116-128	intraluminal suture		Brain Res 2002;931:68-73
Sumii T, et al.	SHR/Taconic Farms	NA	NA	185±6 (SEM)	blood clots embolism	LDF	Stroke 2002;33:831-836
Jin J, et al.	SHR/Hos, Japan SLC	NA	130-160	NA	pMCAO	AR	Pharmacology 2002;64:119-125
Higuchi T, et al.	SHR/Charles River Jpan	NA	261±32	88±12	dMCAO+ipsi-CCAO (Brint)	LDF	J Cereb Blood Flow Metab 2002;22:71-79
Ito T, et al.	SHR/Taconic Farms	8 wk	190-240	SBP=153±6 (SEM)	dMCAO		Stroke 2002;33:2297-2303
Wallace JA, et al.	SHR*	NA	280-320	NA	intraluminal suture		J Cereb Blood Flow Metab 2002;22:1303-1310
Komitova M, et al.	SHR/Mollegaard	NA	NA	NA	dMCAO		J Cereb Blood Flow Metab 2002;22:852-860
Johanson BB, et al.	SHR/Mollegaard	3 mo	NA	NA	dMCAO		J Cereb Blood Flow Metab 2002;22:89-96
Dijkhuizen RM, et al.	SHR/Taconic Farms	NA	300-350	NA	blood clots embolism	MRI	Stroke 2002;33:2100-2104
Yao H, et al.	SHR/Kyushu	5-7 mo	346-444	196 (mean)	photothrombotic dMCAO/Re	LDF	Neuroreport 2002;13:1005-1008
Kitayama J, et al.	SHR/Kyushu	5-8 mo	305-438	161±5 (SEM)	photothrombotic dMCAO	LDF	Brain Res 2001;922:223-228
Furuya K, et al.	SHR*	NA	NA	NA	dMCAO+ipsi-CCAO (Brint)		Stroke 2001;32:2665-2674
Takagi K, et al.	SHR/Funabashi Farm Japan	15-16 wk	280-340	177±11	pMCAO (Tamura)	LDF	Neurol Res 2001;23:662-668
Cole DJ, et al.	SHR*	16-20 wk	375-425	126±9 / 131±7	intraluminal suture		Can J Anaesth 2001;48:807-814
Yan Y, et al.	SHR/Charles River USA	NA	270-300	133±8	intraluminal suture		J Cereb Blood Flow Metab 2001;21:711-721
Rao VLR, et al.	SHR/Charles River USA	NA	250-300	116±11	intraluminal suture	LDF	J Cereb Blood Flow Metab 2001;21:945-954
Zhao Z, et al.	SHR/Charles River Canada	NA	200-250	127±14	dMCAO+ipsi-CCAO	LDF	Brain Res 2001;909:46-50
Marks L, et al.	SHRSP/Gla	3-5 mo	262±11 (SEM)	123±9 (SEM)	dMCAO		Hypertension 2001;38:116-122
Gregersen R, et al.	SHR/Mollegaard	NA	300-335	NA	pMCAO		Exp Brain Res 2001;138:384-392
Yao H, et al.	SHR/Kyushu	5-7 mo	350-438	187 (mean)	photothrombotic dMCAO	LDF	Brain Res Mol Brain Res 2001;91:112-118
Tejima E, et al.	SHR*	NA	250-300	122±3 (SEM)	blood clots embolism	LDF	Stroke 2001;32:1336-1340
Lavie G, et al.	SHR/Tel Aviv	13 wk	NA	127±11	dMCAO		Brain Res 2001;901:195-201
Furuya K, et al.	SHR/Charles River USA	13-16 wk	NA	140±7	pMCAO (Tamura)		J Cereb Blood Flow Metab 2001;21:226-232
Holtz ML, et al.	SHR/Harlan	NA	250-350	108±15	dMCAO+ipsi-CCAO (Brint)		Brain Res 2001;898:49-60
Fredduzzi S, et al.	SHR/Charles River Italy	12-14 wk	250-330	NA	pMCAO (Tamura)		Neurosci Lett 2001;302:121-124
Rao VLR, et al.	SHR/Charles River USA	NA	250-300	NA	intraluminal suture	LDF	J Neurosci 2001;21:1876-1883
Negrin CD, et al.	SHRSP/Gla	13 wk	NA	SBP=186±6 (SEM)	dMCAO		Hypertension 2001;37:391-397
Babu GN, et al.	SHR*	NA	250-300	NA	intraluminal suture	LDF	Neurosci Lett 2001;300:17-20
Legos JJ, et al.	SHR/Taconic Farms	NA	300-350	NA	dMCAO		Brain Res 2001;892:70-77
Slivka AP, et al.	SHR*	NA	240-400	NA	dMCAO+ipsi-CCAO (Brint)		Exp Neurol 2001;167:166-172
Barone FC, et al.	SHR/Taconic Farms	NA	290-340	141±3 (SEM)	dMCAO		J Pharmacol Exp Ther 2001;296:312-321
Relton JK, et al.	SHR/Taconic Farms	NA	250-350	168±15 (SEM)	intraluminal suture		Stroke 2001;32:199-205
Ooboshi H, et al.	SHR/Kyushu	5-7 mo	340-430	167±6 (SEM)	photothrombotic dMCAO	LDF	Stroke 2001;32:1043-1047
Dijkhuizen RM, et al.	SHR/Taconic Farms	NA	300-350	NA	blood clots embolism	LDF / MRI	J Cereb Blood Flow Metab 2001;21:964-971

*substrain unknown; NA, not available. Values are mean ± S.D. BW, body weight; MABP, mean arterial blood pressure;

MCAO, middle cerebral artery occlusion; d, distal; p, proximal; ipsi, ispsilateral; Re, reperfusion;

CBF, cerebral blood flow; LDF, laser Doppler flowmetry; AR, autoradiography; MIR, magnetic resonance imaging.

In SHRSP/Izm, blood pressure was higher, and infarct volume after MCAO was larger than SHR/Izm [27]. However, we found the survival rate after MCAO was lower in SHRSP/Izm than SHR/Izm (87% vs. 99%) in our recent experience. In most experiments with focal ischemia, regular SHR are enough for the preclinical testing of stroke therapies and elucidating the pathophysiology of cerebral ischemia. Consequently, SHRSP were not frequently used for focal ischemia models (9 of 118 [8%]).

**Figure 3:**
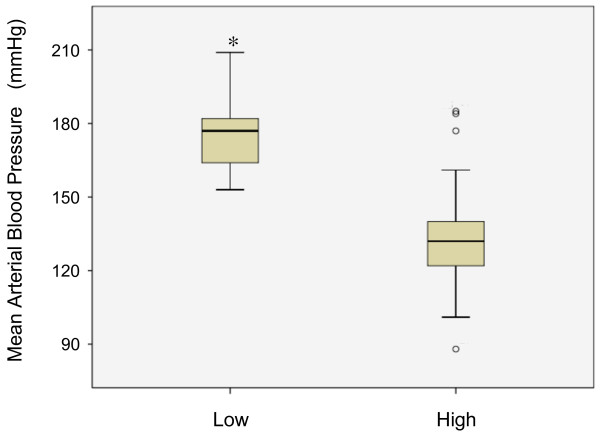
** Box and whisker plot of resting mean arterial blood pressure in experiments with low and high concentrations of anesthesia.** The box shows median value and 25th and 75th percentiles. *P=0.000 for difference in mean arterial blood pressure between the groups (Mann–Whitney u-test).

Resting blood pressure values were available in 62 (53%) articles, while we could not find a description of blood pressure in 56 (47%) articles. The information on blood pressure of SHR is apparently lacking in a substantial amount of literature. Of 35 experiments with standard inhalation anesthesia, rats were intubated, and the anesthesia was maintained with low concentrations of halothane or isoflurane in 13 experiments, while high concentrations of anesthesia were used in 22 experiments often with a facemask in the spontaneously breathing animal. Resting MABP levels were significantly lower (median 132, interquartile range [IQR] 122–140) in those with high concentrations of anesthesia than those (median 177, IQR 164–182) with low concentrations of anesthesia (Mann–Whitney u-test, p = 0.000) (Figure 3). There is general agreement that hypotension during acute stroke is detrimental to perfusion of ischemic brain and tissue outcome [60]. CBF autoregulation was lost when CBF fell below 30% of normal tissue [61]. Arterial blood pressure had a greater influence on CBF in moderately ischemic brain tissue, suggesting potential benefits from raising and possible harm from lowering intraischemic blood pressure. Zhu and Auer (1995) studied the effects of halothane-induced hypotension on histological outcome after transient intraluminal suture occlusion in Wistar rats [62]. Intraischemic hypotension significantly increased infarct size, and the penumbra, defined here as the zone with selective neuronal necrosis, was lost at a blood pressure of 40 mmHg. In line with these observations, mechanical ventilation was required to obtain controlled experimental conditions in focal brain ischemia: spontaneously breathing animals, anesthetized with chloral hydrate i.p. or with halothane via facemask, exhibited respiratory acidosis and decreased blood pressure, resulting in high mortality and a significantly increased infarct volume [63]. 

In SHR, blood pressure levels are classified into 3 time points as follows: (1) the prehypertensive period (4 weeks), (2) the period of rapidly rising blood pressure (8 and 12 weeks), and (3) sustained hypertension (16–18 weeks) [64]. Blood pressure of SHR rises between 1 and 5 months, and blood pressure as well as body weight remain constant thereafter [65]. Survival rate of male SHR/Kyushu declines sharply after 15 months of age. Infarct volume in the young SHRSP (10–12 weeks) was substantially smaller than that in adult SHRSP (5 months) [66]. On the basis of these observations, SHR should be used at 5 to 7 months and SHRSP at 5 months for adult models of focal ischemia. Age as well as body weight should be described in the manuscript to better understand the effects of age on ischemic insults in SHR. We analyzed a possible chronological variability of physiological variables in adult male SHR/Izm (5–7 months old, n = 195) used between 2005 and 2010 for our stroke research, and found that resting MABP in 2009–2010 (163 ± 9 [S.D.] mmHg) was significantly lower than those in 2005–2006 and 2007–2008 (175 ± 10 mmHg and 173 ± 9 mmHg, respectively) (ANOVA and Scheffe test, p = 0.000), while the mean values of body weight were fairly constant (unpublished observation). This observation suggests that MABP could fluctuate even in the closed colony of SHR/Izm under strict monitoring of blood pressure. Physiological parameters of SHR – notably blood pressure, arterial gases, and brain as well as systemic temperature - should be closely monitored and regulated in each experiment. The monitoring of brain temperature, reflected by temporalis muscle (or skull) temperature, would be recommended even in closed skull models such as the intraluminal thread model, because studies that used only rectal temperature measurements may be confounded by unknown variations in brain temperature [67].

Sex differences influence both stroke mechanism and response to therapy [24,68,69]. However, female SHR were only rarely used (2 of 118) (Table 1).

Among the 118 experiments mentioned above, 58 studies tested the efficacy of neuroprotective strategy (Table 2). We assessed the quality of these studies according to published criteria by Macleod et al. [70] with slight modification. These criteria were: (1) peer-reviewed publication, (2) compliance with animal welfare regulations, (3) use of anesthetic without significant intrinsic neuroprotective activity, (4) statement of control of physiological variables, (5) statement of control of brain/head temperature, (6) random allocation to treatment or control, (7) blinded induction of ischemia, (8) blinded assessment of outcome, (9) sample size calculation, and (10) statement of potential conflict of interests. Since statements on conflict of interest have only recently appeared in the pre-clinical stroke literature (2008–2010), this item was not included in the total score, and each study was given a quality score out of a possible total of 9 points. The median quality score was 5.0 (IQR of 4.0-6.0), which was apparently higher than the 3.5 reported by Macleod et al. [70] (experiments published between 1998 and 2003) even considering the minor differences in criteria between the studies, suggesting improved quality of experiments in recent years after the Stroke Therapy Academic Industry Roundtable (STAIR) recommendations [71] and a series of meta-analyses of studies of experimental stroke [70,72,73]. The STAIR-defined criteria for the development and improved clinical testing of neuroprotective drugs are: adequate dose–response data, definition of the time window, blinded and physiologically controlled reproducible studies, hisotological and functional outcomes assessed acutely and long-term, and testing in both permanent and transient MCAO models. High socres (≧6) were largely due to 3 criteria: use of anesthetic without significant intrinsic neuroprotective activity, statement of control of physiological variables, and statement of control of brain/head temperature. A power or sample size calculation was given only in 4 of 58 studies. “Blinded” induction of ischemia or assessment of outcome was described in 8 studies, 7 of which scored high points (≧6). After complete randomization, blinded assessment should be followed for good laboratory practice. Quality of studies with lower scores (≦4) (21 of 58 [36%]) were considered to be inadequate by today’s standards. In addition to the criteria mentioned above, we propose that mortality rate after MCAO surgery should be described. 

**Table 2:** Quality of neuroprotective studies

**Author**	**Year**	**(1)**	**(2)**	**(3)**	**(4)**	**(5)**	**(6)**	**(7)**	**(8)**	**(9)**	**(10)**	**score**
		**peer reviewed**	**copmliance with**	**use of anesthetics**	**physiological**	**brain/head**	**random allocation**	**blinded induction**	**blinded assessment**	**sample size**	**statement**	**SUM of**
		**publication**	**animal welfare**	**without protection**	**variables**	**temperature**	**to Tx or control**	**of ischemia**	**of outcome**	**calculation**	**of COI**	**1~9**
Fujiwara N, et al.	2009	1	1	1	1	0	1	1	0	1	0	**7**
Drummond JC, et al.	2005	1	1	1	1	1	1	0	1	0	0	**7**
Yan Y, et al.	2001	1	1	1	1	1	1	0	1	0	0	**7**
Ito H, et al.	2010	1	1	1	1	1	1	0	0	0	0	**6**
Ishikawa E, et al.	2009	1	1	1	1	1	1	0	0	0	0	**6**
Yamashita T, et al.	2009	1	1	1	1	1	1	0	0	0	0	**6**
Kumai Y, et al.	2007	1	1	1	1	1	1	0	0	0	0	**6**
Zhang B, et al.	2006	1	1	1	0	1	1	0	1	0	0	**6**
Kamiya T, et al.	2005	1	1	1	1	1	1	0	0	0	0	**6**
Ooboshi H, et al.	2005	1	1	1	1	1	1	0	0	0	0	**6**
Takada J, et al.	2005	1	1	1	1	1	1	0	0	0	0	**6**
Kumai Y, et al.	2004	1	1	1	1	1	1	0	0	0	0	**6**
Vemuganti R, et al.	2004	1	1	1	1	1	1	0	0	0	0	**6**
Ren Y, et al.	2004	1	1	1	1	1	1	0	0	0	0	**6**
Sadanaga-Akiyoshi F, et al.	2003	1	1	1	1	1	1	0	0	0	0	**6**
Yan Y, et al.	2003	1	1	1	1	1	1	0	0	0	0	**6**
Sakakibara Y, et al.	2002	1	1	1	1	0	1	0	1	0	0	**6**
Kitayama J, et al.	2001	1	1	1	1	1	1	0	0	0	0	**6**
Furuya K, et al.	2001	1	0	1	1	1	1	0	1	0	0	**6**
Rao VLR, et al.	2001	1	1	1	1	1	1	0	0	0	0	**6**
Furuya K, et al.	2001	1	1	1	1	1	1	0	0	0	0	**6**
Rao VLR, et al.	2001	1	1	1	1	1	1	0	0	0	0	**6**
Slivka AP, et al.	2001	1	1	1	1	0	1	0	1	0	0	**6**
Oyama N, et al.	2010	1	1	1	1	0	1	0	0	0	0	**5**
Sun L, et al.	2010	1	1	1	1	0	1	0	0	0	1	**5**
Ashioti M, et al.	2009	1	1	1	0	0	1	0	0	1	0	**5**
Liu Y-P, et al.	2009	1	1	1	1	0	1	0	0	0	0	**5**
Murata Y, et al.	2008	1	1	1	1	0	1	0	0	0	1	**5**
Matsuzaki T, et al.	2008	1	1	1	1	0	1	0	0	0	0	**5**
Korde AS, et al.	2007	1	1	0	1	1	1	0	0	0	0	**5**
Tureyen K, et al.	2007	1	1	1	1	0	1	0	0	0	0	**5**
Adibhatla RM, et al.	2005	1	1	1	1	0	1	0	0	0	0	**5**
Carswell HV, et al.	2004	1	1	1	1	0	1	0	0	0	0	**5**
Asahi M, et al.	2003	1	1	1	1	0	1	0	0	0	0	**5**
Pettigrew LC, et al.	2003	1	1	0	1	1	1	0	0	0	0	**5**
Takagi K, et al.	2001	1	1	1	1	0	1	0	0	0	0	**5**
Zhao Z, et al.	2001	1	1	1	1	0	1	0	0	0	0	**5**
Zhao X, et al.	2010	1	1	0	1	0	1	0	0	0	1	**4**
Porritt MJ, et al.	2010	1	0	1	0	0	1	0	0	1	1	**4**
Omura-Matsuoka E, et al.	2009	1	1	1	0	0	1	0	0	0	1	**4**
Kozak W, et al.	2008	1	1	1	0	0	1	0	0	0	0	**4**
Zhang B, et al.	2004	1	1	1	0	0	1	0	0	0	0	**4**
Nurmi A, et al.	2004	1	1	0	1	0	1	0	0	0	0	**4**
Wiessner C, et al.	2003	1	1	1	0	0	1	0	0	0	0	**4**
Leker RR, et al.	2002	1	1	0	0	0	1	0	0	1	0	**4**
Jin J, et al.	2002	1	1	1	0	0	1	0	0	0	0	**4**
Lavie G, et al.	2001	1	1	0	0	0	1	0	1	0	0	**4**
Relton JK, et al.	2001	1	1	1	0	0	1	0	0	0	0	**4**
Lammer A, et al.	2006	1	1	0	0	0	1	0	0	0	0	**3**
Gunther A, et al.	2005	1	1	0	0	0	1	0	0	0	0	**3**
Teichner A, et al.	2003	1	1	0	0	0	1	0	0	0	0	**3**
Yamakawa H, et al.	2003	1	1	0	0	0	1	0	0	0	0	**3**
Ito T, et al.	2002	1	1	0	0	0	1	0	0	0	0	**3**
Fredduzzi S, et al.	2001	1	1	0	0	0	1	0	0	0	0	**3**
Barone FC, et al.	2001	1	1	0	0	0	1	0	0	0	0	**3**
Kranz A, et al.	2010	1	0	0	0	0	1	0	0	0	0	**2**
Leker RR, et al.	2002	1	0	0	0	0	1	0	0	0	0	**2**
Legos JJ, et al.	2001	1	0	0	0	0	1	0	0	0	0	**2**

Despite the progress in experimental stroke models, however, the recent angiotensin-receptor blocker candesartan for treatment of acute stroke trial (SCAST) failed to show any beneficial effects in elderly patients with acute stroke and raised blood pressure [74], whereas partial lowering of the blood pressure during reperfusion was beneficial in young SHR [75]. Age may be a factor that accounted for the discrepancy between the pre-clinical animal experiments and the clinical trial in aged patients. Furthermore, besides the inevitable differences between the animal experiments and human stroke, the most critical point of the disparity in the SCAST scenario was that the animal model mimicked the situation of large vessel occlusion, while a substantial portion of the patients in the SCAST study had the cerebral small vessel disease: 29% had lacunar syndrome and 14% hemorrhagic stroke. It is unlikely that a neuroprotective drug acts on lacunar stroke or brain hemorrhage the same as it does on embolic stroke. The mechanistic aspects of the therapeutic strategy should not be left out of accounts of either animal or human studies, since different mechanisms of protection between experimental and clinical settings may lead to failure in translational research.

### Cerebral blood flow

Among the 118 articles (2001–2010) on Table 1, CBF was determined in 60 experiments: 56 with laser-Doppler flowmetry, 3 with autoradiography, and 3 with magnetic resonance imaging. One study with autoradiography also adopted 2-dimensional laser-Doppler perfusion imaging (Moor Instruments, Inc.), and in another study both magnetic resonance imaging and laser-Doppler flowmetry were used. One of the major advantages of the magnetic resonance imaging method was that this noninvasive modality provided additional information such as diffusion-weighed imaging [76,77] and hemorrhagic transformation [77] in addition to perfusion imaging. The power of CBF autoradiography lies in its ability to quantitate the local CBF in small discrete brain regions, although flow can be assessed only once and sacrifice of the animal is required. With regard to focal ischemia models, the golden standard of CBF measurement is the quantitative 3-dimensional autoradiography [46]. Laser-Doppler flowmetry is an easy method to monitor a relative measure of blood perfusion, and is commonly used in small animals. Practical considerations in this method include confounding effects such as superfluous light, heterogeneous distribution of superficial blood vessels, and movement artifacts. In the studies of focal ischemia, one-point CBF measurement with laser-Doppler flowmetry may show no significant (a false negative) difference in CBF after MCAO between the groups. To overcome this issue, we determined CBF with the laser-Doppler flowmetry “scanning” method as previously described [66]: a laser-Doppler flowmetry probe was laterally scanned, and CBF of the distal MCA territory was measured at five points (2 mm posterior and 2.0, 2.5, 3.0, 3.5, and 4.0 mm lateral to the bregma), and 5 CBF values in one rat were transformed to an area under curve according to the trapezoidal rule. This “scanning” method worked well, but it is time-consuming and requires much effort. Two-dimensional cortical CBF mapping is possible with laser-Doppler perfusion imaging, which is a sophisticated and robust image registration [78,79]. This system, however, requires an expensive set-up.

### Beyond substrains

To overcome the genetic heterogeneity in substrains of SHR and SHRSP, several approaches are currently in progress. In order to establish a system to facilitate the systematic collection of rat strains and genetic characterization, the National BioResource Project-Rat (NBRP-Rat) was launched in 2002 [80]. By the end of 2008, more than 500 rat strains, including substrains of SHR and SHRSP, had been deposited in the NBRP-Rat. With this database, researchers can select a rat strain (e.g., SHR/Izm) to instantly compare its genetic background against all rat strains typed at NBRP-Rat [81]. Such a phenotypic ‘Strain Ranking’ allows visual data scoring, which provides an opportunity to easily and simultaneously compare phenotypic values for multiple rat strains. This database could in theory be applied to find genetic differences between the substrains with a different phenotype. Needless to say, this approach will not immediately resolve the major heterogeneity between strains, but a huge rat repository is a reasonable attempt.

Another approach is the congenic strategy based on the genome-wide linkage or quantitative trait loci (QTL) analysis to examine the effects of polygenic trait on the stroke phenotype. Jeffs et al. identified 3 major QTL on chromosomes 1, 4, and 5 related to stroke sensitivity or large infarct size in the SHRSP after MCAO by performing a genome scan in an F2 cross of SHRSPGla and WKYGla [82]. Recently, a novel gene-targeting technology utilizing zinc-finger nucleuses, which does not rely on using species-specific embryonic stem cell lines, has been proved to work successfully even in rats to generate a knockout of specific DNA regions [83]. However, this method is not applicable to the search for new genetic traits in SHR or SHRSP, because the genes or specific DNA regions for essential hypertension or stroke have not been identified in either animals or human beings. Almost all common diseases including stroke are based on polygenes. Stroke falls within the category of complex traits, arising from numerous gene-gene and gene-environment interactions [84,85]. To overcome this complexity, the role of identified QTL on stroke susceptibility is currently studied through the production of congenic lines: one or several QTL can be replaced with those of the control strain to examine functional evidence for the role of genes distinct between the two strains. For example, a blood pressure QTL on rat chromosome 1 was introgressed from WKY/Izm to SHRSP/Izm by repeated backcrossing to cancel out the heterogeneity in genetic background between the strains. This congenic removal of the blood pressure QTL increased collateral CBF after MCAO, and despite a small decrease in blood pressure, demonstrated a substantial reduction in infarct volume after MCAO compared with SHRSP/Izm, showing a beneficial effect beyond blood pressure [66]. Congenic substrains are used to narrow down the implicated congenic segment to aid the identification of positional candidate genes [86]. As Aitman et al. showed, microarray combined with congenic strategy may lead to the identification of a causative gene once congenic strains containing smaller QTL have been produced [87]. The congenic strategy would be particularly useful to investigate the substrain differences in SHR and SHRSP or stroke proneness in the future.

## Concluding comments

We have reviewed the early development of various focal ischemia models in substrains of SHR, and summarized recent reports on this topic. Although SHR, which have comorbidity (i.e., spontaneous hypertension), are suitable for stroke research, only 11% of animal experiments on neuroprotection involved testing in hypertensive rats [30]. Distal MCAO without carotid occlusions in SHR is best suited for the penumbra concept. To translate the results in animal models into human stroke, common mechanisms underlying the ischemic injury or protection by therapy need to be elucidated both in the animal model and human stroke. One of the confounding factors is that genetic heterogeneity among substrains of SHR and SHRSP may make the situation complicated. Therefore, we attempted to provide an overview of focal ischemia models in diverse substrains of SHR. Spin-off findings from recent studies were that age, body weight, and even blood pressure of SHR were not indicated in a number of articles. We would like to emphasize the importance of these basic physiological parameters in light of the different phenotypes among substrains of SHR.

## Competing interests

The authors declare that they have no competing interest.

## Authors’ contributions

HY conducted the literature search and wrote manuscript. TN is responsible for critically revising for intellectual content. Both authors read and approved the final manuscript.
